# Dual Key Speech Encryption Algorithm Based Underdetermined BSS

**DOI:** 10.1155/2014/974735

**Published:** 2014-05-14

**Authors:** Huan Zhao, Shaofang He, Zuo Chen, Xixiang Zhang

**Affiliations:** ^1^School of Information Science and Technology, Hunan University, Changsha, Hunan 410082, China; ^2^Science College, Hunan Agricultural University, Changsha, Hunan 410128, China

## Abstract

When the number of the mixed signals is less than that of the source signals, the underdetermined blind source separation (BSS) is a significant difficult problem. Due to the fact that the great amount data of speech communications and real-time communication has been required, we utilize the intractability of the underdetermined BSS problem to present a dual key speech encryption method. The original speech is mixed with dual key signals which consist of random key signals (one-time pad) generated by secret seed and chaotic signals generated from chaotic system. In the decryption process, approximate calculation is used to recover the original speech signals. The proposed algorithm for speech signals encryption can resist traditional attacks against the encryption system, and owing to approximate calculation, decryption becomes faster and more accurate. It is demonstrated that the proposed method has high level of security and can recover the original signals quickly and efficiently yet maintaining excellent audio quality.

## 1. Introduction

As speech communications in our daily life become more and more common, the importance of providing a high level of security is sharply increasing. For that reason, a series of speech encryption methods have been proposed. Among which, the analogue encryption is one of the most popular encryption techniques widely used in speech communication. Generally, there are four categories of cryptographic algorithms in speech communication: frequency-domain scrambling (e.g., the frequency inverter and the band splitter), time-domain scrambling (e.g., the time element scrambling), amplitude scrambling (also known as the masking technique that covers the speech signal by the linear addition of pseudorandom amplitudes), and two-dimensional scrambling that combines the frequency-domain scrambling with the time-domain scrambling [1, 2]. In addition, there are many other analogue speech encryption algorithms in the transform domain, for example, discrete cosine transform, fast Fourier transform, wavelet transform, and so forth [3–5]. Up to date, many new speech encryption algorithms including BSS-based [2, 6], chaotic cryptosystem [7–10], and encryption using circulant transformations [11] have been developed. Due to the fact that the great amount data of speech communications and real-time communication has been required, it is not suitable to utilize traditional encryption methods directly for speech communication encryption. As such, to explore speech encryption methods that have a high level of security and efficiency and high speed in decryption while retaining excellent audio quality is an urgent issue.

Blind source separation (BSS) is used to recover unknown source or signals that are independent mutually of their observed mixtures without knowing the mixing coefficients. So, it is also known as independent component analysis (ICA). Recently, signals encryption has been more applied on the image cryptosystems [12–14] but less on the speech encryption. Speech encryption method based BSS, of which the security dependent on the difficulty of solving the underdetermined BSS problem where the number of the observed mixed signals is less than that of the source signals. The sufficient condition for constructing the underdetermined mixing matrix for encryption is presented based on the source inseparability of BSS [15].

One-time pad [16] is a simple and completely unbreakable symmetric cipher, and it has two perfect characteristics: the key is random and has the same length as the message. The key space is large enough to resist brute-force attacks as long as the key is long enough. So, the message is secure as long as the key is protected. Motivated by the randomness and initial conditions' sensitivity of chaotic signals, we present an underdetermined BSS-based dual key speech encryption scheme in this paper. The dual key of which are random signals (one-time pad) and chaotic signals, namely, key signals I and II, respectively. The main purpose of the algorithm is to mask the original speech signals by mixing the original speech with key signals I and II. In the decryption process, approximate calculation method is used to recover the original speech signals. The underdetermined blind source separation is a significant challenge in blind source separation (BSS) where the number of the source signals is greater than that of the mixed signals. In addition, the using of the key signals I and II (one-time pad and chaotic signal) ensures high security of the algorithm. Both extensive computer simulations and performance analysis results show that the proposed method has high level of security while retaining excellent audio quality.

The rest of this paper is organized as follows. In Section 2, firstly, we introduce the BSS mixing model and the underdetermined BSS problem briefly, secondly, the details of speech encryption and decryption are described, and finally, we analyze the feasibility of approximate calculation in the decryption process. Sections 3 and 4 conduct computer simulations to illustrate and analyze the performance of the method. We conclude this paper in Section 5.

## 2. Proposed Method

### 2.1. BSS Mixing Model and Underdetermined Problem [2]

Suppose that *s*_1_(*t*), *s*_2_(*t*),…,*s*_*M*_(*t*) is *M* independent source signals and *N* observed mixtures of the source signals are *x*_1_(*t*), *x*_2_(*t*),…,*x*_*N*_(*t*)  (*M* ≪ *N*). The linear BSS mixing model is represented as follows:

(1) $$\begin{array}{c} x\left(t\right)=A_{e}s\left(t\right), \end{array}$$

where *s*(*t*) = [*s*_1_(*t*), *s*_2_(*t*),…,*s*_*M*_(*t*)]^*T*^, which is *M* × 1 column vector collected from the source signals, similarly, *N* × 1 column vector *x*(*t*) = [*x*_1_(*t*), *x*_2_(*t*),…,*x*_*N*_(*t*)]^*T*^ collects the observed signals, and *A* is an *N* × *M* mixing matrix that contains the mixing coefficients. The aim of BSS is to find a *M* × *N* demixing matrix *W* such that output vector:

(2) $$\begin{array}{c} u\left(t\right)=Wx\left(t\right)=WAs\left(t\right)=PDs\left(t\right), \end{array}$$

where *P* ∈ *R* ^*M*×*M*^ is a permutation matrix and *D* ∈ *R* ^*M*×*M*^ is a diagonal scaling matrix. When the number of the mixed signals is less than that of the source signals; that is, *M* > *N*, BSS becomes a difficult case of the underdetermined problem, in which the complete separation of the source signals is impossible.

### 2.2. Encryption

**Figure 1:**
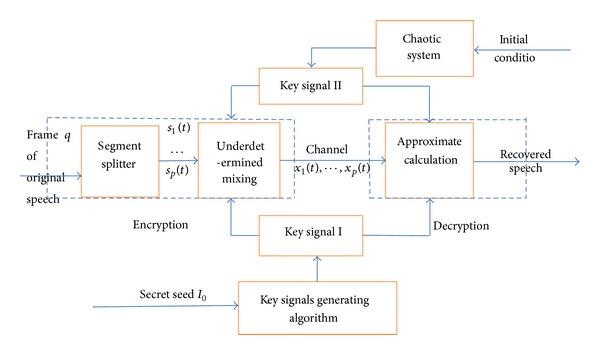
Block diagram of underdetermined BSS-based double key speech encryption.

The main idea of the proposed algorithm is to construct the intractable underdetermined BSS problem in encryption, and in decryption it can only be solved with the dual key. The block diagram of the underdetermined BSS-based speech encryption scheme is shown in Figure 1.

Two main steps in the encryption process are the segment splitter and the underdetermined mixing. Suppose that the original speech is divided into frames, and every frame is encrypted, respectively; *q* is the frame pointer. The frame *q* is encrypted as follows.

(1) Segment splitter: the segment splitter first partitions the frame *q* into *P* segments *s*_1_(*t*), *s*_2_(*t*),…,*s*_*p*_(*t*), *t* = 1,…, *T*, where *T* is the segment length.
(2) Underdetermined mixing: the source signals are composed of three parts, they are original speech signals, key signals I generated by pseudorandom number generator (PRNG) with secret seed *I*_0_, and key signals II from chaotic system. *s*(*t*) = [*s*_1_(*t*), *s*_2_(*t*),…,*s*_*p*_(*t*)]^*T*^ denotes original speech, key signals I are *k*(*t*) = [*k*_1_(*t*), *k*_2_(*t*),…,*k*_*p*_(*t*)]^*T*^, and *h*(*t*) = [*h*_1_(*t*), *h*_2_(*t*),…,*h*_*p*_(*t*)]^*T*^ is key signals II. Therefore, 3*p* × 1 column vector of the source signals is [*s* ^*T*^(*t*), *k* ^*T*^(*t*), *h* ^*T*^(*t*)]^*T*^. A *p* × 3*p* underdetermined mixing matrix *A*_*e*_ = [*B* *αB* *βB*] for encryption is first generated randomly, where *B* is a *P* × *P* matrix of full rank, which is pseudorandomly generated with normal distribution between −1 and 1, 1 ≪ *α*, *β* ≪ 2 are scalar values to make the original speech be covered well by the dual key signals. The encryption equation can be represented as follows:
  (3) x(t)=Ae[sT(t),kT(t),hT(t)]T=[BαBβB](s(t)k(t)h(t))=Bs(t)+αBk(t)+βBh(t),
  where *x*(*t*) = [*x*_1_(*t*), *x*_2_(*t*),…,*x*_*p*_(*t*)]^*T*^ is the observed signals. (Parameters *P*, *T*, secret seed *I*_0_, initial condition of chaotic system, and scalar *α*, *β* are inserted into the head data of the encryption speech in a definite format for transmission.)

### 2.3. Decryption

Once the mixture signals *x*(*t*) = [*x*_1_(*t*), *x*_2_(*t*),…,*x*_*p*_(*t*)]^*T*^ are received, the key signals I are regenerated by the secret seed *I*_0_ and the key signals II are produced by the chaotic system using the initial conditions. Usually, BSS is then performed [2, 17] to recover original signals. But in this paper, we employ approximate calculation to recover original signals.

#### 2.3.1. The Approximate Calculation for Decryption

Multiply *k* ^*T*^(*t*) at both sides of (3), and we get equation:

(4) $$\begin{array}{c} x\left(t\right)k^{T}\left(t\right)=Bs\left(t\right)k^{T}\left(t\right)+\alpha Bk\left(t\right)k^{T}\left(t\right)+\beta Bh\left(t\right)k^{T}\left(t\right). \end{array}$$

Similarly, multiply *h* ^*T*^(*t*) at both sides of (3), and then get equation

(5) $$\begin{array}{c} x\left(t\right)h^{T}\left(t\right)=Bs\left(t\right)h^{T}\left(t\right)+\alpha Bk\left(t\right)h^{T}\left(t\right)+\beta Bh\left(t\right)h^{T}\left(t\right) \end{array}$$

denoted by

(6) $$\begin{array}{c} R_{xk}=x\left(t\right)k^{T}\left(t\right),\;\;R_{sk}=s\left(t\right)k^{T}\left(t\right),\\ R_{kk}=k\left(t\right)k^{T}\left(t\right),\;\;R_{hk}=h\left(t\right)k^{T}\left(t\right),\\ R_{xh}=x\left(t\right)h^{T}\left(t\right),\;\;R_{sh}=s\left(t\right)h^{T}\left(t\right),\\ R_{kh}=k\left(t\right)h^{T}\left(t\right),\;\;R_{hh}=h\left(t\right)h^{T}\left(t\right). \end{array}$$

Equations (4) and (5) can be represented by (7) and (8), respectively,

(7) $$\begin{array}{c} R_{xk}=BR_{sk}+\alpha BR_{kk}+\beta BR_{hk}, \end{array}$$

(8) $$\begin{array}{c} R_{xh}=BR_{sh}+\alpha BR_{kh}+\beta BR_{hh}. \end{array}$$

Since the original speech signals are independent statistically of the key signals I and II, we have *R*_*sk*_ ≪ *R*_*kk*_,  *R*_*sh*_ ≪ *R*_*hh*_, that is, *BR*_*sk*_ ≪ *αB* *R*_*kk*_,  *BR*_*sh*_ ≪ *βB* *R*_*hh*_,  1 ≪ *α*, *β* ≪ 2; therefore, (7) and (8) are represented approximately by (9) and (10):

(9) $$\begin{array}{c} R_{xk}\approx \alpha BR_{kk}+\beta BR_{hk}, \end{array}$$

(10) $$\begin{array}{c} R_{xh}\approx \alpha BR_{kh}+\beta BR_{hh}. \end{array}$$

Plus (9) and (10), we can get equation:

(11) $$\begin{array}{c} {R_{xk}+R}_{xh}\approx B\left(\alpha R_{kk}+\beta R_{hk}+\alpha R_{kh}+\beta R_{hh}\right). \end{array}$$

So we obtain an estimate for *B* as follows:

(12) $$\begin{array}{c} \hat{B}=\left(R_{xk}+R_{xh}\right){\left(\alpha R_{kk}+\beta R_{hk}+\alpha R_{kh}+\beta R_{hh}\right)}^{-1}. \end{array}$$

Substituting *B* in (3) with (11), original signals *s*(*t*) can be estimated as

(13) s^(t)=(αRkk+βRhk+αRkh+βRhh) ×(Rxk+Rxh)−1x(t)−αk(t)−βh(t).

#### 2.3.2. The Steps of Calculating Original Signals for Decryptions

Parameters *P*, *T* and scalar *α*, *β* are transmitted together with the secret seed *I*_0_ and initial condition of chaotic system in the head data of the encryption speech. The original speech signals can be decrypted with very high quality by employing the decryption equation (13). Supposing that the mixed signals are received and the double key signals are regenerated, the original signals can be calculated as follows:

- calculate *R*_*xk*_, *R*_*kk*_, *R*_*xh*_, *R*_*hk*_, *R*_*kh*_, *R*_*hh*_ respectively;
- calculate (*αR*_*kk*_ + *βR*_*hk*_ + *αR*_*kh*_ + *βR*_*hh*_) and (*R*_*xk*_ + *R*_*xh*_)^−1^;
- calculate $\hat{s}(t)$ using (13).

#### 2.3.3. Analysis of the Approximate Calculation for Decryption

Key signals I and II are generated by pseudorandom number generator (PRNG) and chaotic system, respectively. They are both independent statistically of the original signals. For illustrating the feasibility of the approximate calculation, we compute values of *R*_*sk*_, *R*_*kk*_, *R*_*sh*_, and *R*_*hh*_ in the example. The original signals of digital “1” in English are regarded as a frame, which is divided into two segments, that is, *P* = 2, *T* = 16000, the original signals *s*(*t*) = [*s*_1_(*t*), *s*_2_(*t*)]^*T*^, correspondingly key signals I and II are *k*(*t*) = [*k*_1_(*t*), *k*_2_(*t*)]^*T*^, *h*(*t*) = [*h*_1_(*t*), *h*_2_(*t*)]^*T*^. The key signals I are generated by PRNG with the secret seed *I*_0_ = *p* × *T* = 32000, and choosing initial condition (*a*, *b*, *c*) = (35,3, 28), [*h*(1), *y*(1), *z*(1)] = [0,1.001,0], the key signals II (*h*(*t*)/40) are generated by Chen-Lee chaotic system [18]:

(14) h˙=−yz+ah,y˙=hz+by,z˙=13hy+cz.

Finally, we calculate the values of *R*_*sk*_, *R*_*kk*_, *R*_*sh*_, *R*_*hh*_ and get the average values of diagonal elements value of *R*_*sk*_ = *s*(*t*)*k* ^*T*^(*t*), *R*_*kk*_ = *k*(*t*)*k* ^*T*^(*t*), *R*_*sh*_ = *s*(*t*)*h* ^*T*^(*t*), and *R*_*hh*_ = *h*(*t*)*h* ^*T*^(*t*) are 1.3775, 5365.35, −0.5212, and 876.29, respectively.

In the example, the original signals are also splitted into other different numbers of segments, which are *P* = 4, 8, corresponding to *T* = 8000, 4000, use the same key signals I and II, and compute *R*_*sk*_, *R*_*kk*_, *R*_*sh*_, and *R*_*hh*_, respectively.  Table 1 shows the results of the average value of diagonal elements and upper-triangular for comparison of *R*_*sk*_, *R*_*kk*_, *R*_*sh*_, and *R*_*hh*_ in three different cases.

From Table 1 we can see that the average values of diagonal elements of *R*_*sk*_ are considerably much smaller than those of *R*_*kk*_, and the average values of upper-triangular elements of *R*_*sh*_ are also much smaller than those of *R*_*hh*_, and obviously, *R*_*sk*_ ≪ *R*_*kk*_, *R*_*sh*_ ≪ *R*_*hh*_; therefore, using approximate calculation for decryption is feasible, which means that the decryption method in this paper not only have the characteristic of computing simply and quickly but also maintaining excellent audio quality.

## 3. Computer Simulations

**Figure 2:**
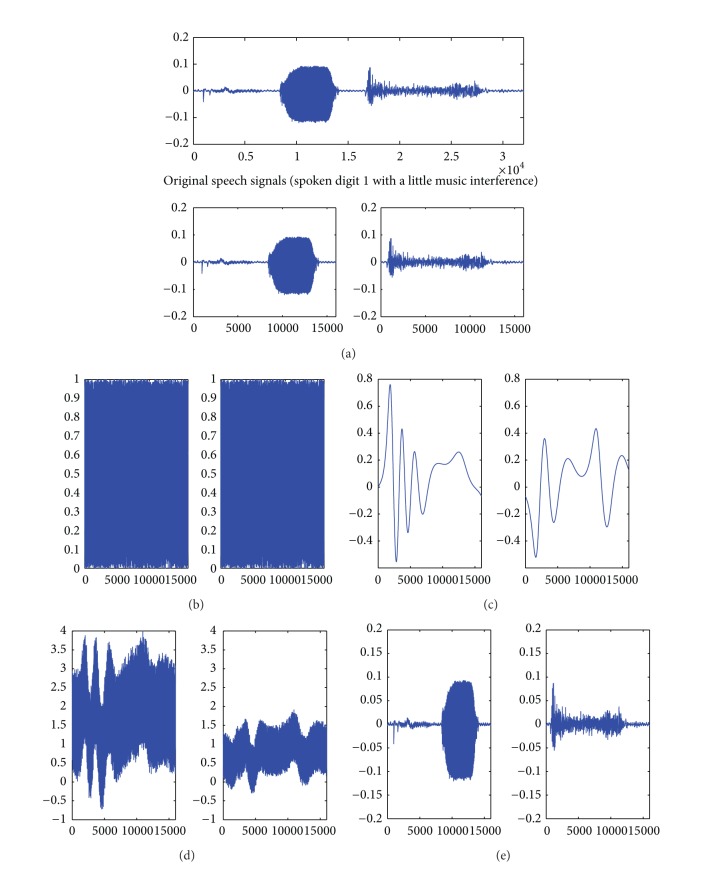
(a) The original speech signals and two segments *s*_1_(*t*), *s*_2_(*t*); (b) two segments of key signals I *k*_1_(*t*), *k*_2_(*t*); (c) two segments of key signals II *h*_1_(*t*), *h*_2_(*t*); (d) two encrypted segments *x*_1_(*t*), *x*_2_(*t*); (e) two recovered segments by approximate calculation ${\hat{s}}_{1}\left(t\right)$, ${\hat{s}}_{2}\left(t\right)$.

In order to illustrate the feasibility of the proposed scheme, we carry out extensive computer simulation. In common experiments, recorded audio files in wave format are adopted and transmitted within local area network. In our experiment, we use the speech file recording a man saying the digit “1” in English. The speech signals are sampled at 16 KHz, as shown in Figure 2(a). For the purpose of simplifying experiment process, we regard the speech signals as one frame directly and separate them into two segments; that is, *p* = 2, *T* = 16000, as shown in Figure 2(a). The key signals I are generated by PRNG with the secret seed *I*_0_ = *p* × *T* = 32000, and Figure 2(b) is the split wave of key signals I. Choosing initial condition (*a*, *b*, *c*) = (35,3, 28) and [*h*(1), *y*(1), *z*(1)] = [0,1.001,0], the key signals II (*h*(*t*)/40) are generated by Chen-Lee chaotic system [18]:

(15) $$\begin{array}{c} \dot{h}=-yz+ah,\\ \dot{y}=hz+by,\\ \dot{z}=\frac{1}{3}hy+cz, \end{array}$$

and the split wave of which is showed in Figure 2(c). Choosing *α* = *β* = 2, the underdetermined mixing matrix *A*_*e*_ = [*B* *αB* *βB*] used for simulation is

(16) Ae=(0.95010.60680.23110.48601.90031.21370.46230.97201.90031.21370.46230.9720)

using (3), and two cipher texts are deduced quickly.  Figure 2(d) shows the two cipher-text segments. Obviously, the original speech signals are well covered with the mixed sets of key signals I and II. In the decryption process, the mixed signals are received and the double key signals are regenerated; we can regain the original speech signals according to the steps of approximate calculation decryption method. The recovered signals are showed in Figure 2(e).

## 4. Performance Analysis

### 4.1. Signal-to-Noise Ratio Computation

For the purpose of quantifying the performance of the proposed method, we calculated the signal-to-noise ratio (SNR) index of original signals segments in each encrypted signal segments and decrypted signals segments. Particularly, the SNR index of original segments in the decrypted segments is represented as follows:

(17) $$\begin{array}{c} \text{SNR}\left(\text{dB}\right)=10\log\left\{\frac{{\sum_{t=0}^{T}\left[s\left(t\right)-\hat{s}(t\right)]}^{2}}{\sum_{t=0}^{T}s^{2}\left(t\right)}\right\}, \end{array}$$

where *s*(*t*) is original signals and $\hat{s}(t)$ is decrypted original signals that are calculated by the approximate calculation method. If $\hat{s}(t)$ is replaced by *x*(*t*), which denotes encrypted signals, we can obtain the SNR index of encrypted signals. Employing the data in computer simulations, we can get SNR of two original signals segments in two encrypted segments and two decrypted signals segments.  Table 2 shows the results.

These SNR indexes show that in the encrypted segments dual key signals have well masked the original segments, and the original signals in the decrypted segments that are recovered by approximate calculation method have excellent quality.

### 4.2. Security Analysis

We take advantage of the underdetermined BSS problem to propose a dual key encryption algorithm in this paper. There are three aspects to ensure the security of the algorithm. Firstly, the intractability of the underdetermined BSS problem can be ensured by the mixing matrix for encryption. Secondly, the key signals I that as long as the original speech signals have the prefect property of the one-time pad cipher, which is statistically independent and non-Gaussian characteristics.

**Figure 3:**
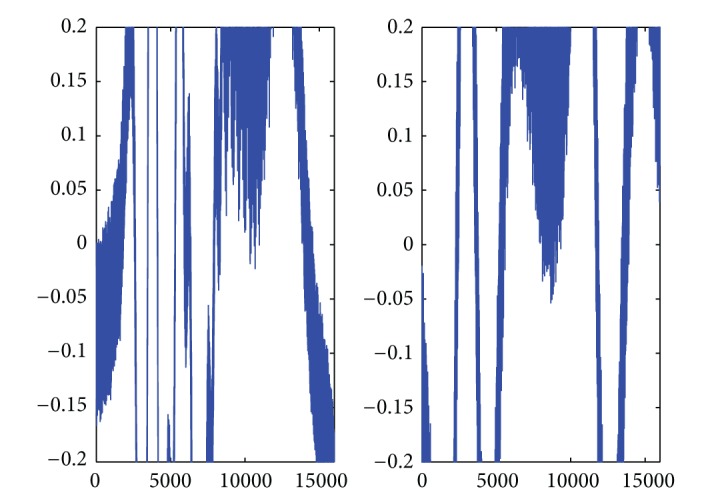
Decrypted signals of the proposed method with mismatch of secret keys.

Finally the key signals II are generated from the Chen-Lee chaotic system that has the characteristic of power randomness and high sensitivity to initial condition. In order to illustrate the sensitivity of the proposed encryption algorithm to secret keys, we choose an estimate of the initial condition that is (*a*, *b*, *c*) = (35, −3,28), [*h*(1), *y*(1), *z*(1)] = [0.001,1, 0.001], in which there is a slight mismatch with the real initial condition and use the same key signals I with secret seed *I*_0_ = 32000.  Figure 3 shows two segments of the recovered signals utilizing the approximate calculation for decryption. Obviously, the recovered signals with the wrong secret key are totally different from the original speech signals. In short, the proposed method is sensitive to secret keys and immune against the ordinary attacks on cryptosystems, for example, cipher-text-only attack, known-plaintext attack, chosen-plaintext attack, and brute-force attract.

## 5. Conclusions

In this paper, we proposed a new dual key encryption scheme based on the underdetermined BSS problem. Since the mixing matrix for encryption ensures the intractability of the underdetermined BSS problem, and the key signals I approximately have the prefect property of the one-time pad cipher, and the key signals II are generated from the chaotic system that has the characteristic of power randomness and high sensitivity to initial condition. In the decryption process, using approximate calculation decryption method can recover the original signals quickly and efficiently yet maintaining high level audio quality. The design of this encryption method has five merits: (1) it is impossible to recover the original signals without the parameters in the head data of the encryption speech signals; (2) the approximate calculation method used in decryption process ensures the recovery of the original signals efficiently yet maintaining excellent speech quality; (3) the key signals I approximately has the prefect property of the one-time pad cipher, in which the length of the key is the same as the original speech signals; hence, the space of the keys is so large that all brute-force attacks against the system are infeasible; (4) the key signals II generated from chaotic system that provides the present scheme having the property of cipher text are very sensitive to secret keys, and (5) it can resist all kinds of traditional attacks against cryptosystems.

## Figures and Tables

**Table 1:** Comparison of average values of diagonal elements and upper triangular elements for R_*sk*_, *R*_*kk*_, *R*_*sh*_, and *R*_*hh*_.

Segment	Average values of diagonal elements				Average values of upper triangular elements			
	R_*kk*_	R_*sk*_	R_*hh*_	R_*sh*_	R_*kk*_	R_*sk*_	R_*hh*_	R_*sh*_
*p* = 2, *T* = 16000	5365.35	1.3775	876.29	−0.5212	4914.5	1.4676	488.4	0.087
*p* = 4, *T* = 8000	2682.7	0.6888	438.15	−0.2606	2281.4	0.5392	163.1	0.170
*P* = 8, *T* = 4000	1343.96	0.0645	219.08	−0.1303	1092.6	0.1002	57.99	0.051

**Table 2:** SNR (dB) of two original signals segments in two encrypted segments and two decrypted signals segments.

Original signals segments	Encrypted signals segments		Decrypted signals segments	
	*x*_1_	*x*_2_	${\hat{s}}_{1}$	${\hat{s}}_{2}$
*s*_1_	81.4021	65.5826	−94.595	1.0563
*s*_2_	105.586	89.7596	25.2364	−101.7182
